# Exposure to Electronic Cigarettes Impairs Pulmonary Anti-Bacterial and Anti-Viral Defenses in a Mouse Model

**DOI:** 10.1371/journal.pone.0116861

**Published:** 2015-02-04

**Authors:** Thomas E. Sussan, Sachin Gajghate, Rajesh K. Thimmulappa, Jinfang Ma, Jung-Hyun Kim, Kuladeep Sudini, Nicola Consolini, Stephania A. Cormier, Slawo Lomnicki, Farhana Hasan, Andrew Pekosz, Shyam Biswal

**Affiliations:** 1 Department of Environmental Health Sciences, Johns Hopkins University, Bloomberg School of Public Health, Baltimore, Maryland, United States of America; 2 Children′s Research Foundation Institute, University of Tennessee Health Science Center, 50 N. Dunlap, Memphis, Tennessee, United States of America; 3 Department of Environmental Sciences, Louisiana State University, Baton Rouge, Louisiana, United States of America; 4 Department of Chemistry, Louisiana State University, Baton Rouge, Louisiana, United States of America; 5 W. Harry Feinstone Department of Molecular Microbiology and Immunology, Johns Hopkins University, Bloomberg School of Public Health, Baltimore, Maryland, United States of America; Albany Medical College, UNITED STATES

## Abstract

Electronic cigarettes (E-cigs) have experienced sharp increases in popularity over the past five years due to many factors, including aggressive marketing, increased restrictions on conventional cigarettes, and a perception that E-cigs are healthy alternatives to cigarettes. Despite this perception, studies on health effects in humans are extremely limited and *in vivo* animal models have not been generated. Presently, we determined that E-cig vapor contains 7x10^11^ free radicals per puff. To determine whether E-cig exposure impacts pulmonary responses in mice, we developed an inhalation chamber for E-cig exposure. Mice that were exposed to E-cig vapor contained serum cotinine concentrations that are comparable to human E-cig users. E-cig exposure for 2 weeks produced a significant increase in oxidative stress and moderate macrophage-mediated inflammation. Since, COPD patients are susceptible to bacterial and viral infections, we tested effects of E-cigs on immune response. Mice that were exposed to E-cig vapor showed significantly impaired pulmonary bacterial clearance, compared to air-exposed mice, following an intranasal infection with *Streptococcus pneumonia*. This defective bacterial clearance was partially due to reduced phagocytosis by alveolar macrophages from E-cig exposed mice. In response to Influenza A virus infection, E-cig exposed mice displayed increased lung viral titers and enhanced virus-induced illness and mortality. In summary, this study reports a murine model of E-cig exposure and demonstrates that E-cig exposure elicits impaired pulmonary anti-microbial defenses. Hence, E-cig exposure as an alternative to cigarette smoking must be rigorously tested in users for their effects on immune response and susceptibility to bacterial and viral infections.

## Introduction

Electronic cigarettes (E-cigs) have grown rapidly in popularity since first coming to the US market in 2007. The E-cig market for 2013 was approximately $1.7 billion, and US sales of E-cig products are expected to surpass tobacco products in a decade. Recent surveys indicate that use of E-cigs by adults in the US quadrupled between 2009 and 2010 [1], and is currently at 3.4% [2]. More than 700,000 individuals have switched from traditional cigarettes to electronic cigarettes. Although E-cigs were originally marketed as smoking cessation aids, E-cig use is also gaining popularity among never smokers, who now constitute a substantial portion of the E-cig market. Use among teenagers doubled between 2011 and 2012 [3], with an estimated 1.78 million middle and high school students using E-cigs, which suggests that E-cigs are an emerging public health issue.

While cigarette smoke contains more than 5,000 different chemicals, E-cig vapor consists of relatively few constituents. The primary ingredient in E-cig vapor is a stabilizing agent, typically propylene glycol and/or glycerol, which is vaporized through delivery of an electric current to the E-cig cartridge. These vapor droplets carry nicotine and added flavors, such as menthol, piña colada, and strawberry. Because E-cigs do not produce any combustion products or tar, the perception remains, both among the general public and many physicians, that E-cigs are indeed a safer alternative to conventional cigarettes. Analyses of E-cig vapor have identified many different chemicals that could be potentially toxic or carcinogenic, including particulates, formaldehyde, nitrosamines, metals, carbonyls, volatile organic compounds, and polycyclic aromatic hydrocarbons; although these compounds are generally reported to be lower than in cigarette smoke [4–9]. Additionally, the levels of several of these toxicants are increased after vaporization, due to heat and/or voltage generated by the battery [10]. A recent *in vitro* study revealed that exposure of lung epithelial cells to E-cig-conditioned medium induces a gene expression signature similar to tobacco smoke [11]. However, human studies on effects of E-cig exposure are limited to very short-term responses, and there are no *in vivo* animal studies. Based on current information, the Food and Drug Administration (FDA), the World Health Organization (WHO), and the European Respiratory Society have indicated that electronic cigarettes are not considered a safe alternative to smoking. Furthermore, recent studies indicate that E-cigs may not be an effective therapy for smoking cessation [12].

Cigarette smoke contains 10^14^ free radicals/puff [13] and causes oxidative damage, inflammation and apoptosis, which drive the pathogenesis of chronic obstructive pulmonary disease (COPD). More importantly, cigarette smoke exposure impairs innate and adaptive immune responses against bacteria and viruses, which predispose to COPD exacerbations, a major contributor to COPD-related morbidity, mortality and health care costs [14]. The goal of our current study was to develop a murine model of E-cig exposure and to assess the pulmonary defenses against bacterial or viral infection. We demonstrated that E-cig vapor is surprisingly a rich source of free radicals, and E-cig exposure resulted in airway inflammation, oxidative stress, and impaired anti-bacterial and anti-viral responses that include increased bacterial burden and viral titers in lungs, impaired bacterial phagocytosis, and increased virus-induced morbidity and mortality. This impaired anti-microbial response indicates that E-cig exposure is not a safe alternative to cigarette smoking.

## Methods

### Animals

Male C57BL/6 (age 8 wks) were purchased from NCI Frederick. All mice were housed under controlled conditions for temperature and humidity in a specific pathogen free facility, using a 12 h light/dark cycle. Mice were randomly assigned to each treatment group. All experimental protocols were performed in accordance with the standards established by the US Animal Welfare Acts, as set forth in NIH guidelines and in the Policy and Procedures Manual of the Johns Hopkins University Animal Care and Use Committee. All procedures were approved by the Johns Hopkins University Animal Care and Use Committee. Animals were monitored daily, and any mice exhibiting signs of distress were euthanized. Mice were anesthetized via inhalation of isofluorane, and all animals were euthanized by inhalation of carbon dioxide followed by exsanguination.

### Animal exposure

Mice were exposed to NJOY menthol bold (1.8% nicotine) rechargeable E-cigs, except where indicated. A subset of mice was exposed to NJOY traditional bold (1.8% nicotine) rechargeable E-cigs. E-cig vapor was generated using the Jaeger-Baumgartner CSM 2080 (CH Technologies), in which a 2 s puff (35ml) was taken every 10 s and mixed with dilution air via a pump set at 1.05 L/min. Mice were exposed via a whole-body exposure system for 1.5 h, twice per day for 2 wks. Exposure level was observed in real time via a MicroDust Pro (Casella CEL), and exposure was assessed by measuring serum cotinine at 1 h after exposure. The smoke machine has a maximum capacity of 30 E-cigs that can be loaded onto a carousel that rotates at 1 RPM. For each exposure, we loaded 6 E-cigs, which were evenly spaced so that each E-cig was puffed for 2 s, once per minute, for a total of 6 puffs per minute. E-cig batteries were charged prior to each exposure and E-cig cartridges were replaced each week. The lifespan of each cartridge varied considerably, so they were also replaced whenever the real-time exposure monitor detected a decrease in output of a specific E-cig. The nephelometer readings averaged 1613±165 mg/m^3^ throughout the exposures, although it should be noted that the nephelometer was not calibrated for E-cig vapor. Thus, these readings were simply used to maintain consistent exposure rather than to quantify the exposure. Air-exposed mice were exposed to filtered air at the same flow rate as E-cig exposed mice.

### Serum cotinine

Serum cotinine was measured via the Cotinine Direct ELISA Kit (BQ Kits, Inc) as recommended by the manufacturer. Blood was collected from the tail vein of mice (N = 5) either within 1 h of the final exposure or 24 h after exposure. Serum cotinine after exposure to E-cigs was similar to reports of serum cotinine from human E-cig users. Cotinine returned to baseline levels at 24h after exposure.

### Electron paramagnetic resonance (EPR)

Total particulate matter (TPM) from E-cigs was collected on a Cambridge filter pad by vaping the E-cig using a CSM-SSM single cigarette smoking machine (CH Technologies), as described above. Fifty puffs yielded 30.4 mg of TPM that was collected on the filter. After collection of particulate matter over 50 puffs, the filter was immediately transferred to the EPR tube for measurement of the paramagnetic signal. EPR spectra were measured using a Bruker EMX 10/2.7 EPR spectrometer (X-band) equipped with a dual cavity utilizing modulation and microwave frequencies of 100 kHz and 9.516 GHz, respectively. Parameters for radical signal measurement were: 2.05 mW power; modulation amplitude of 4.0 G; scan range of 100 G; time constant of 40.96 msec corresponding to a conversion of 163.84 ms; sweep time of 167.77 seconds; receiver gain 3.56x10^4^; and three scans. After collection of the spectrum, a native background signal of the filter pad was subtracted to obtain the final spectrum. Radical concentrations were calculated by comparing the signal peak area, as calculated from the ΔH_p-p_ multiplied by the relative intensity, to a DPPH standard. The g-factors were determined by the WinEPR program.

### Inflammation

Lungs were lavaged three times with 1 ml PBS, and total cells were stained with AccuStain and counted via the ADAM-MC automated cell counter (Digital Bio) (N = 10). Differential cell counts were determined by cytospin preps stained with H&E and determined based on standard cytological criteria. IL-6, IFNγ, TNFα, IL-17A, MCP-1, and MIP-2 were measured by ELISA (R&D Systems). For surface expression of CD36 and MARCO, alveolar macrophages were stained with CD36-APC and MARCO-PE antibodies and surface expression was quantified by flow cytometry.

### Bacterial infection and clearance

At 1 h after the final exposure, mice were anesthetized with isofluorane and infected with 1x10^5^ CFU of *S*. *pneumoniae* via intranasal instillation [15]. After 24 h, mice were euthanized by CO_2_ inhalation, and lungs were either homogenized in 1ml PBS or lavaged two times with 1 ml PBS (N = 10). Serial dilutions were plated on bacterial growth plates and colonies were counted after 24 h. For *ex vivo* infections, alveolar macrophages were harvested from BAL (N = 5) via differential plating and 4x10^4^ macrophages were plated in 96-well plates. *S*. *pneumoniae* was added at multiplicity of infection of either 10 or 20. After 4 h, media was removed and plated on blood agar plates. For quantification of phagocytosis, alveolar macrophages were infected at an MOI of 20 for 1 h, then media was removed, extracellular bacteria were killed with gentamycin (200 μg/ml for 10 min at 37 C), and macrophages were lysed with 0.2% triton X-100 (10 min at room temperature). Extracellular and intracellular fractions were plated on blood agar.

### Viral infection

Anesthetized mice were administered 10^2^ or 10^3^ 50% tissue culture infectious dose (TCID_50_) of mouse adapted influenza A/California/4/2009 H1N1 in 30 ul of DMEM [16]. The mice were monitored daily for weight loss (a measure of morbidity) and mortality (N = 10). At 4 days post infection, a subset of mice (N = 5) was euthanized, lungs were excised and stored at -70C. The mouse lungs were weighed and homogenized as a 10% suspension in DMEM. Infectious virus titers were determined by performing TCID_50_ assay on MDCK cells as previously described [17]. Other subsets of mice were euthanized at day 4 (N = 5) or day 8 (N = 4), and the airways were lavaged to assess inflammation and cytokine levels.

### Statistical analyses

The Student’s two-tailed t-test was used to determine statistical significance between each group. Values are presented as means ± standard error.

## Results

### Model development

**Figure 1:**
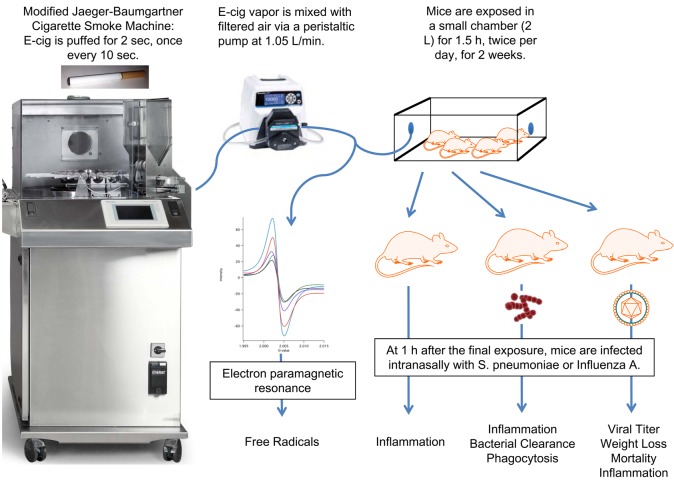
Schematic of our E-cig exposure model.

To generate E-cig vapor, we modified the Jaeger-Baumgartner Cigarette Smoke Machine 2080, which is a mainstream exposure system, for use with E-cigs (Fig. 1). Modifications included disconnecting the lighting element and disabling the cigarette loader and ejector. Due to the relatively short half-life of E-cig vapor, mice were exposed in a small chamber with a high air exchange rate of 2 minutes. E-cigs were puffed according to the Federal Trade Commission (FTC) puffing protocol of 2 s, 35 ml puffs. Previous studies indicate that E-cig users have similar serum cotinine concentrations as cigarette smokers [18, 19], which often exceed 300 ng/ml in heavy smokers, and we designed our exposure protocol to attain comparable cotinine concentrations. E-cig vapor was delivered to the exposure chamber via one 2 s puff every 10s and was mixed with dilution air (1.05 L/min). Mice were exposed to E-cig vapor for 1.5 h, twice per day for 2 weeks. Immediately after the final E-cig exposure, serum cotinine reached 267±17 ng/ml, which is comparable to human E-cig users and smokers. At 24 h after the final exposure, serum cotinine fell to 0.6±0.1 ng/ml, which was similar to cotinine levels observed in air-exposed mice (0.6±0.0 ng/ml).

### Free radical measurements in E-cig vapor

**Figure 2:**
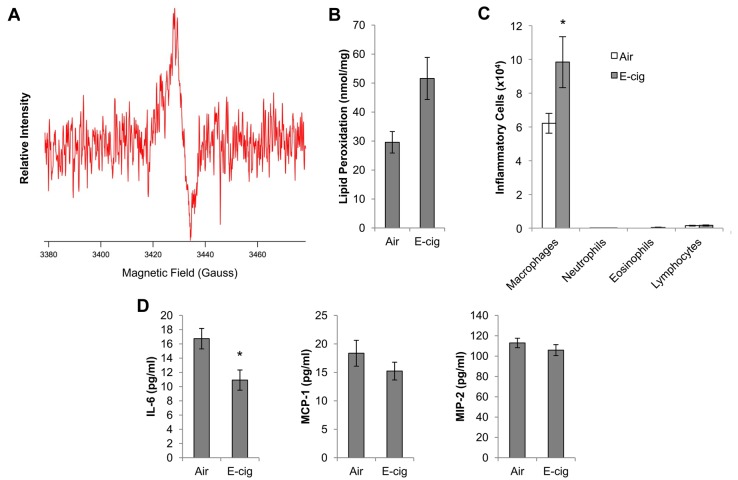
E-cig-induced pulmonary response. (A) EPR spectra of E-cig TPM. The presented spectrum is a result of subtraction of a Cambridge filter pad EPR signal before and after collection of TPM. (B) Lipid peroxidation was measured by thiobarbituric acid reactive substances (TBARS) in lung homogenates from C57BL/6 mice that were exposed to air or E-cig vapor for 1.5 h, twice per day, for 2 wks. (C) Inflammatory cells were quantified in the BAL at 24h after the final exposure. (D) Cytokines were analyzed in cell-free BAL fluid from air and E-cig exposed mice at 24h after the final exposure. N = 10 mice per group. *p<0.05 by Student’s two-tailed t-test.

Free radicals are typically associated with the combustion process. However, to determine whether E-cig vapor contains free radicals, E-cigs were puffed as depicted in Fig. 1, and total particulate matter was analyzed by electron paramagnetic resonance (EPR). Traditional cigarettes produce 2 classes of radicals during the smoking event—gas phase radicals and those associated with TPM. Gas phase radicals are typically very short lived and quench before reaching the lower respiratory tract; and thus their health effects are unclear. On the contrary, TPM-associated radicals are long lived and deposit in all areas reached by TPM. Thus TPM radicals were the focus of this experiment. The EPR spectra demonstrated a free radical concentration of 7x10^11^ spins/puff, with a g-value of 2.0035 and a linewidth (ΔH_p-p_) of 6.8 Gauss (Fig. 2A). Thus, E-cig vapor contains a substantial number of free radicals that could be potentially toxic to cells.

### Effects of E-cig exposure on pulmonary response

To assess the effects of E-cig exposure on the lungs, mice were exposed to air or E-cig vapor for 2 weeks, and oxidative stress was measured in lungs and airway inflammation was quantified in the bronchoalveolar lavage (BAL). Consistent with the detection of free radicals in E-cig vapor, lungs from E-cig exposed mice contained significantly elevated levels of oxidative stress, as assessed by thiobarbituric acid reactive substances (TBARS) (Fig. 2B). E-cig exposure also resulted in a 58% increase in macrophage infiltration (p<0.05), but had no impact on infiltration of neutrophils, eosinophils, or lymphocytes (Fig. 2C). This increase in macrophage influx to the airways is similar to our previous studies that have investigated the inflammation after exposure to cigarette smoke. Thus, a sub-chronic exposure to E-cig vapor induces a modest inflammatory response in the airways.

We also assessed cytokine levels in BAL fluid after air and E-cig exposure. Despite a low concentration of IL-6 in the BAL of air-exposed mice, we detected a significant reduction in IL-6 concentration in E-cig exposed mice (Fig. 2D). Monocyte chemotactic protein 1 (MCP-1) and macrophage inflammatory protein 2 (MIP-2) were similar between air and E-cig exposed mice (Fig. 2D), although they both showed slight decreases after E-cig exposure. Thus, despite increased macrophages in airways at 24h after E-cig exposure, we did not detect increased inflammatory cytokines in the BAL.

### Effects of E-cig exposure on pulmonary anti-bacterial defenses

**Figure 3:**
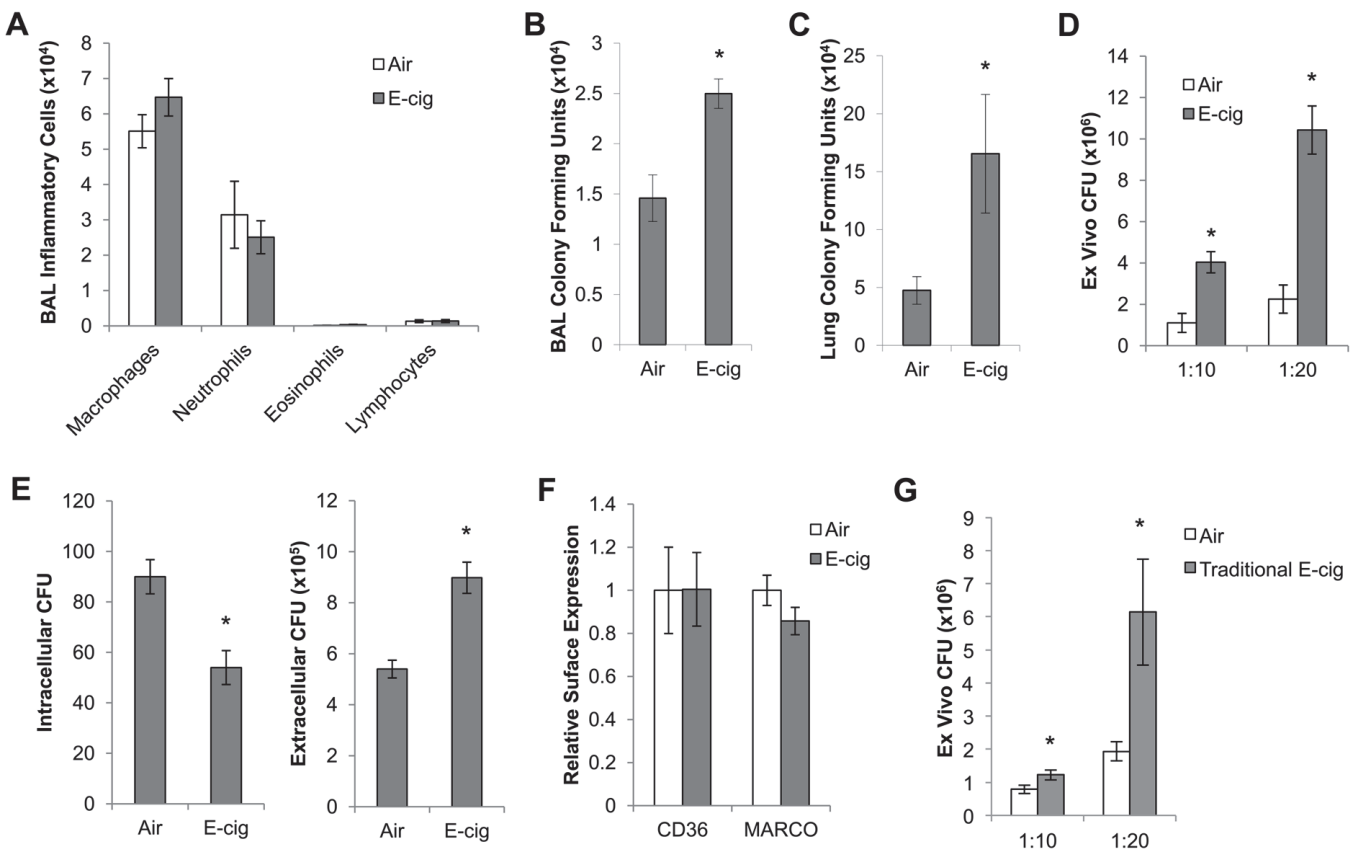
E-cig exposure reduces pulmonary bacterial clearance in mice infected with *S*. *pneumoniae*. Mice were exposed to air or E-cig for 2 wks, then infected intranasally with 1x10^5^ colony forming units (CFU) of *S*. *pneumoniae*. Inflammation (A) and bacterial CFUs (B) were determined in BAL at 24h after infection (N = 10 mice per group). (C) In a separate group of mice, bacterial CFUs were quantified in lung homogenates (N = 10 mice per group). (D) Alveolar macrophages from air or E-cig exposed mice were harvested and infected with *S*. *pneumoniae* at multiplicities of infection of 10 and 20. Bacterial CFUs were quantified in cell-free culture media at 4 h after infection. (E) Intracellular (internalized) and extracellular bacteria (cell-free culture media) were quantified at 1 h after *ex vivo* infection of alveolar macrophages with an MOI of 20. (F) Alveolar macrophages were harvested at 2h after final exposure, stained with antibodies against CD36 and MARCO, and analyzed by flow cytometry. (G) Bacterial clearance was measured in alveolar macrophages from mice exposed to air or traditional E-cig vapor. *p<0.05 by Student’s two-tailed t-test.

To determine whether E-cig exposure alters pulmonary anti-bacterial defense, mice were exposed to air or E-cig vapor for 2 wks, followed by intranasal infection with a 10^5^ inoculum of a clinical isolate of *S*. *pneumoniae*. Bacterial infection resulted in an influx of neutrophils at 24h, which was not significantly altered by E-cig exposure (Fig. 3A). However, relative to the air-exposed group, E-cig exposure resulted in significant increases in pulmonary bacterial burden measured in either bronchoalveolar lavage fluid (Fig. 3B) or lung homogenates (Fig. 3C). To determine whether this impaired anti-bacterial defense was due to a defect in macrophage function, alveolar macrophages from air or E-cig exposed mice were infected *ex vivo* with *S*. *pneumoniae* at multiplicity of infections (MOI) of either 10 or 20. E-cig exposure resulted in significantly increased bacterial burden in culture media at 4 h after infection (Fig. 3D), indicating that E-cig exposure suppresses bacterial clearance by alveolar macrophages. To determine whether this impaired bacterial clearance by macrophages was due to defective phagocytic function, alveolar macrophages from air or E-cig exposed mice were infected *ex vivo* with *S*. *pneumoniae* for 1 h, then extracellular and intracellular bacteria were quantified. Macrophages from E-cig exposed mice exhibited significant decreases in internalized bacteria and concurrently, significant increases in extracellular bacteria (Fig. 3E), indicating that E-cig exposure impaired bacterial phagocytosis by alveolar macrophages. Phagocytosis is often mediated by scavenger receptors on the cellular surface [20]; however E-cig exposure did not alter surface expression of CD36 or MARCO, as determined by flow cytometry (Fig. 3F). Lastly, to determine whether this impaired anti-bacterial defense was specific to menthol E-cig exposure, we measured *ex vivo* bacterial clearance by alveolar macrophages from mice that were exposed to traditional flavor (1.8% nicotine) E-cigs. Similar to exposure to menthol E-cigs, traditional E-cigs induced a 34% increase in inflammation (p<0.05) (data not shown), and alveolar macrophages from these mice exhibited significantly impaired clearance of bacteria (Fig. 3G), indicating that this response was not specific to mentholated E-cigs. Thus, E-cig exposure resulted in impaired anti-bacterial defenses, including defective bacterial phagocytosis, leading to enhanced bacterial propagation.

### Effects of E-cig exposure on pulmonary anti-viral defense

**Figure 4:**
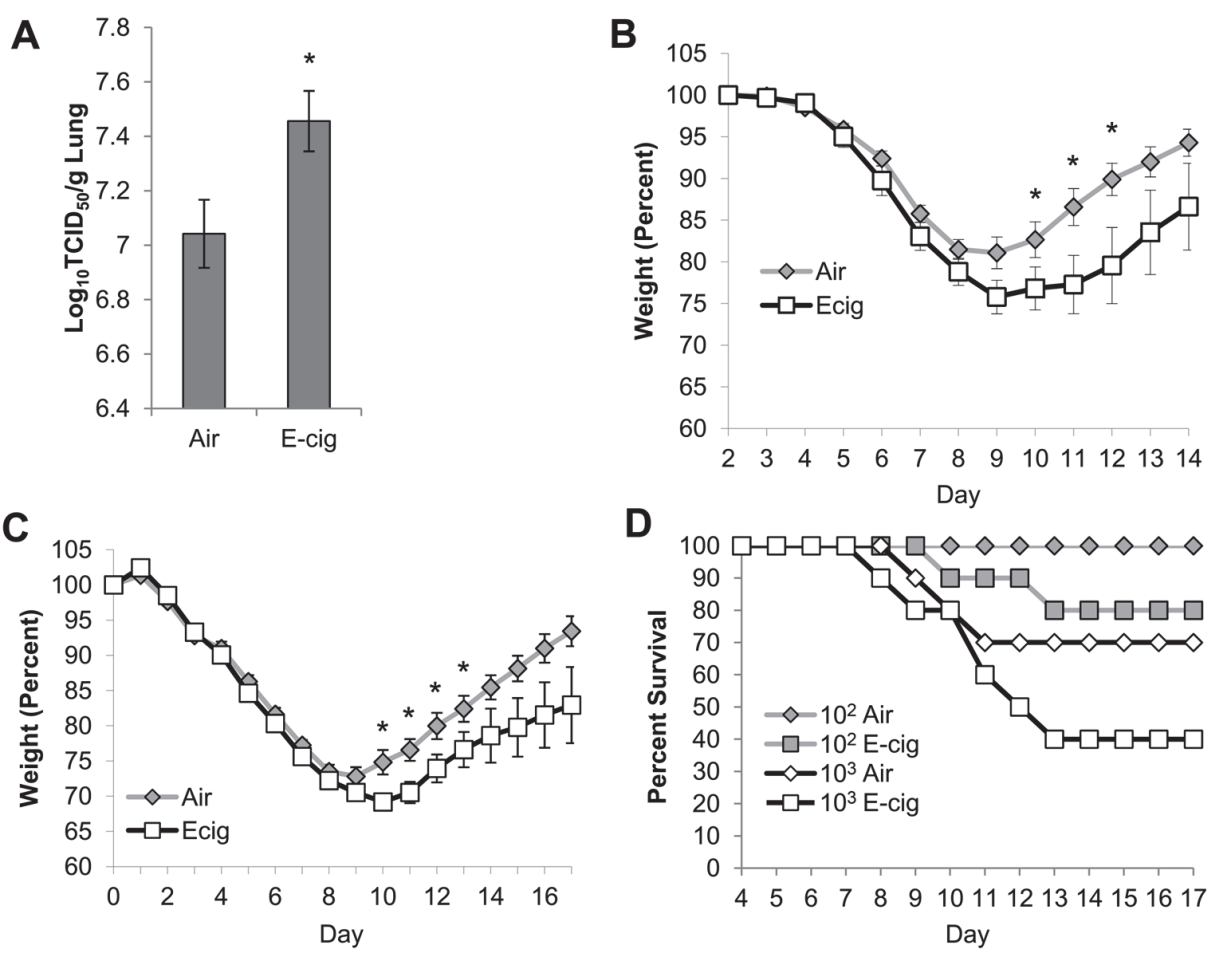
E-cig exposure impairs viral clearance and causes significant morbidity and mortality in mice following influenza virus infection. Mice were exposed to air or E-cig for 2 wks, then infected intranasally with either TCID_50_ 10^2^ (A-B) or TCID_50_ 10^3^ (C) of H1N1 virus. (A) Viral titer was determined by TCID_50_ assay in lung homogenates at 4 days after infection (N = 5 mice per group). (B-C) Mice were weighed daily after infection with either TCID_50_ 10^2^ (B) or 10^3^ (C), and values are presented as percent of starting weight (N = 10 mice per group). For mice that died during the experiments, body weights were included in the analysis up until the day of death. (D) Mortality curves in response to intranasal infection with TCID_50_ 10^2^ or 10^3^ H1N1 (N = 10 mice per group). *p<0.05 by Student’s two-tailed t-test.

To quantify the effects of E-cig exposure on susceptibility to viral infection, mice were exposed to air or E-cig vapor for 2 wks then infected intranasally with 10^2^ TCID_50_ of a mouse-adapted H1N1 influenza virus. Quantification of viral titer in lungs of mice at day 4 revealed significantly elevated infectious virus titers in mice that had been exposed to E-cig vapor prior to infection, compared to mice that had been exposed to air (Fig. 4A). Weight loss was used as an indicator of illness, and mice were weighed daily for 2 wks after infection. Initial weight loss was similar between air and E-cig exposed mice, which peaked at day 9 (Fig. 4B). However, air-exposed mice began to recover at day 10, while recovery was delayed in E-cig exposed mice. E-cig exposure resulted in significant reductions in percent weight at days 10–12, and surprisingly also caused death in 20% (2 of 10) of the E-cig exposed mice (Fig. 4D), while none of the air exposed mice died. To further assess the effects of E-cig exposure on virus-induced mortality, mice were infected with a higher dose of virus (10^3^ TCID_50_). Infection with this higher dose resulted in rapid weight loss in both air and E-cig exposed groups, beginning at day 2 and peaking at days 9–10. Once again, initial weight loss was similar between both groups (Fig. 4C); however, recovery was delayed in mice that were exposed to E-cig vapor prior to infection, compared to mice that were exposed to air. E-cig exposure resulted in significant reductions in percent weight at days 10–13 compared to air controls, and E-cig exposure also increased mortality from 30% to 60% (Fig. 4D) (p = 0.0947 by log-rank test). Taken together, E-cig exposure reduces anti-viral defenses and increases virus-induced morbidity and mortality.

**Figure 5:**
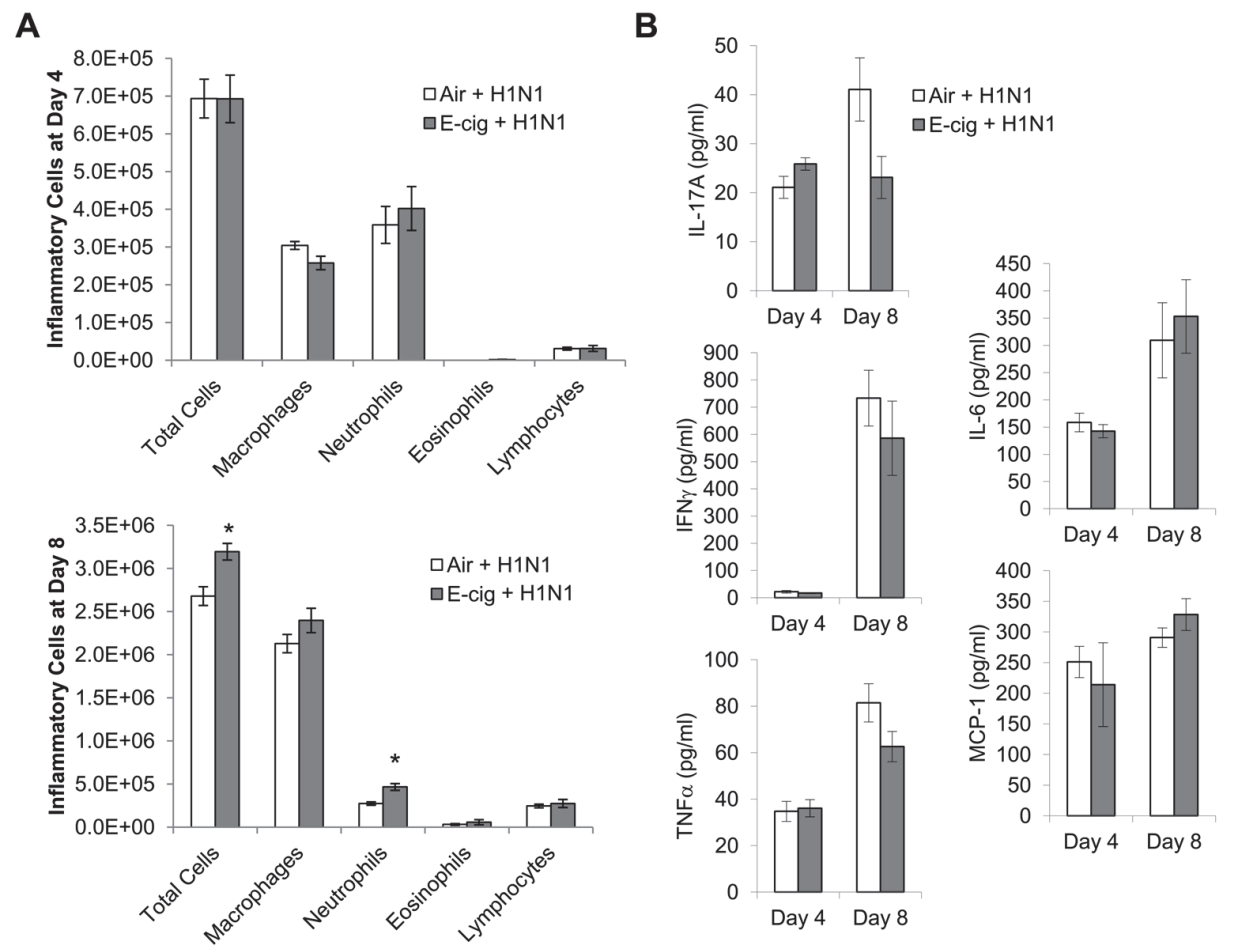
Influenza-induced inflammation is altered by E-cig exposure. Mice were exposed to air or E-cig for 2 wks, then infected intranasally with TCID_50_ 10^2^ of H1N1. BAL was collected at day 4 (N = 5) and day 8 (N = 4) after infection, followed by quantification of inflammatory cells (A) and cytokines (B). *p<0.05 by Student’s two-tailed t-test.

To further characterize the virus-induced morbidity, we assessed airway inflammation in response to influenza infection (10^2^ TCID_50_) just prior to the onset of illness (day 4) as well as near the peak of illness (day 8). In both air and E-cig exposed mice, airway inflammation was substantially higher at day 8 compared to day 4 Fig (5A). We did not observe any significant changes in airway inflammation between air and E-cig exposed mice at day 4; however, at day 8 E-cig exposed mice contained significant increases in total inflammatory cells and neutrophils (Fig. 5A). Comparison of cytokine levels between days 4 and 8 showed significant increases in TNFα and IFNγ in both air and E-cig exposed mice at day 8 (Fig. 5B). Interestingly, IL-17A was significantly increased at day 8 only in air exposed mice, while IL-6 was significantly increased at day 8 only in E-cig exposed mice (Fig. 5B). Direct comparisons between air and E-cig exposed mice did not show any significant differences in cytokine levels; however, T cell-derived cytokines IL-17A and IFNγ tended to be lower in E-cig exposed mice (p = 0.07 and p = 0.34, respectively), while macrophage-derived cytokines MCP-1 and IL-6 tended to be higher in E-cig exposed mice (p = 0.27 and p = 0.66, respectively). Thus, E-cig exposure increased neutrophilic inflammation at day 8 after virus infection, compared to air exposure, but decreased Th1 and Th17 cytokine levels.

## Discussion

Commercially sold E-cigs contain a wide array of nicotine concentrations. Many E-cig users have serum cotinine (a metabolite of nicotine) concentrations that are similar to those of cigarette smokers [18, 19]. To date, clinical effects of E-cig use are limited and there are no established animal models of E-cig exposure. In the present study, we developed a murine model of E-cig exposure. The serum cotinine concentration in E-cig exposed mice, which was 267 ng/ml, is comparable to those levels found in both cigarette smokers and E-cig users, which often exceed 300 ng/ml. Using this model, we have demonstrated that sub-chronic E-cig exposure causes a mild inflammatory response that is characterized by infiltration of macrophages to the airways. This influx in macrophages is similar to the inflammatory response after exposure to cigarette smoke [21, 22]. The adverse pulmonary response in E-cig exposed mice is also consistent with data among E-cig users indicating that E-cig use causes acute pulmonary effects, including increased airway resistance and decreased FeNO [23]. Despite a modest increase in macrophage number, E-cig exposure caused reductions in pro-inflammatory cytokines IL-6 and MCP-1 compared to air-exposed mice. However, since cytokine levels are already low in air-exposed mice, it is not clear whether this E-cig-induced reduction in cytokines will have any biological impact in the absence of other stressors. Thus, although it is possible that E-cig exposure is less harmful to the lungs than cigarette smoke, it is clear that E-cig vapor elicits a pulmonary response.

We and others have shown in experimental and clinical studies that cigarette smoking disrupts pulmonary innate immune defenses and increases susceptibility to bacterial and viral infections [20, 24–26]. Patients with COPD experience multiple viral and bacterial exacerbations of their disease [27], which is the major cause of COPD-related morbidity and mortality. Therefore, our main interest was to determine whether E-cig exposure has any impact on pulmonary immune defenses against bacteria or viruses that are commonly associated with acute exacerbations of COPD. We observed a significant impairment in bacterial clearance in lungs of mice exposed to E-cigs for 2 wks. Although, there was no significant difference in the influx of neutrophils between E-cig and air exposed groups at 24 h after infection, we observed poor bactericidal activity, partly due to defective bacterial phagocytic function by alveolar macrophages harvested from E-cig exposed mice, compared to air controls. We previously demonstrated that mice exposed to cigarette smoke for 1 wk exhibited defective bacterial phagocytosis [20], which was similar to the effects observed here after exposure to E-cig vapor. We also previously showed that cigarette smoke alters surface expression of scavenger receptors that are important in bacterial recognition and uptake [20]; however, E-cig exposure did not alter surface expression of macrophage scavenger receptors, MARCO or CD36.

Among mice infected with *S*. *pneumoniae*, E-cig exposure did not affect macrophage cell number. This was somewhat surprising since E-cig exposure alone resulted in a significant increase in macrophages compared to air exposure. However, this result is consistent with our previous studies in mice exposed to cigarette smoke plus bacteria, which demonstrate that cigarette smoke has substantial effects on bacterial clearance while having only minor effects on inflammatory cell number [20]. This suggests that E-cig exposure, similar to cigarette smoke exposure, impairs the function of inflammatory cells rather than their recruitment. Additionally, while inflammation after E-cig alone (Fig. 2C) was not directly compared to E-cig plus bacteria (Fig. 3A), the number of macrophages was higher in the mice exposed only to E-cig than in mice exposed to E-cig plus bacteria. Previous reports indicate that *S*. *pneumoniae* and other bacteria induce rapid turnover and apoptosis of alveolar macrophages, which peaks at 6 hours after infection, followed by steady increases in macrophage number over the ensuing hours-days [28, 29]. It is not clear what effect E-cig exposure has on macrophage turnover, although it may make the macrophages more susceptible to *S*. *pneumoniae*-induced apoptosis.

E-cig exposure also resulted in enhanced susceptibility to influenza infection, based on increased percent weight loss, mortality, and viral titer. Interestingly, the difference in weight loss and mortality was not evident during the first seven days of infection, but manifested only during resolution of infection. The enhanced susceptibility to viral infection caused by E-cig exposure is consistent with the effects of cigarette smoke exposure on influenza infection, including enhanced viral titer at days 3–4 and impaired resolution of infection [30, 31]. At day 8, E-cig exposure significantly increased airway neutrophils, but reduced the levels of several cytokines, including IL-17A, IFNγ, and TNFα. IL-17 is involved in protective immunity against influenza infection [32]. Both cigarette smoke and nicotine have been shown to inhibit pulmonary T cell responses, including secretion of IFNγ, and enhance susceptibility to virus infection [31, 33], which suggests that exposure to nicotine, such as in E-cigs, may exhibit immunosuppressive effects. However, as we demonstrated, nicotine is rapidly metabolized by the mouse and is not detectable beyond 24h after E-cig exposure. Thus, it is not clear whether activation of cholinergic receptors by nicotine at the time of influenza infection has prolonged effects on the immune response.

Nicotine has many immunosuppressive effects, including impaired antibacterial defenses [34, 35] and altered macrophage activation to suppress adherence, chemotaxis, phagocytosis and intracellular killing of bacteria [36–38]. Thus, it is possible that the impaired antibacterial and antiviral responses observed in E-cig exposed mice are partially mediated by nicotine. However, other components of E-cig vapor may also contribute to altered pulmonary responses. Approximately 75% of E-cig vapor is comprised of propylene glycol and/or glycerol. Inhalation exposure to propylene glycol, which is also found in theatrical fog machines, has been shown to elicit detrimental effects on lung function. Employees who work in close proximity to fog machines have impaired adjusted FEV1 and FVC levels compared to employees working further away [39], and acute exposure to propylene glycol for 1 minute results in significantly reduced FEV1/FVC [40]. Furthermore, a recent case report observed a woman who developed lipoid pneumonia after 7 months of using E-cigs [41]. The patient contained lipid-laden macrophages in the BAL, which the authors attributed to inhalation of glycerol-based E-cig vapor. Lipid-laden macrophages exhibit impaired bacterial phagocytosis [15, 42]. Thus, it is likely that even E-cigs containing 0% nicotine will still alter the pulmonary response. NJOY, the manufacturer of the E-cig used in this study, does not sell E-cigs with 0% nicotine, which precludes us from examining the effects of nicotine in this current study, but future studies will address the role of nicotine and other individual constituents.

Cigarette smoke contains 10^14^ free radicals per puff [13], and is a potent source of oxidative stress. Many free radicals are produced during combustion of tobacco, and since E-cigs do not contain any combustion products, it has been assumed that E-cigs will have very low levels of free radicals. We determined that E-cig vapor contains 7x10^11^ free radicals per puff and elicits a significant increase in oxidative stress. While this concentration is several orders of magnitude lower than in cigarette smoke, it is nonetheless a substantial number of free radicals that could be potentially toxic to cells. This concentration is surprising due to the fact that E-cig vapor does not contain any combustion products. It is possible that the heat and/or current generated during vaporization could induce formation of radicals, and indeed, the battery output voltage has been shown to play a role in generation of other toxic chemicals [10].

While E-cig vapor contains reduced concentrations of many toxicants compared to cigarette smoke and does not contain any combustion products or tar, it remains unclear whether these reduced toxicant levels still produce detrimental pulmonary effects. *In vitro* studies suggest that E-cig liquids produce cytotoxic effects, although the effects may be reduced compared to cigarette smoke extract-induced cytotoxicity [43, 44].

We acknowledge several limitations to this study. Our exposure was designed to model the nicotine concentration of E-cig users; however, reports of serum cotinine concentrations are highly variable, and many reports indicate that nicotine concentration in E-cigs is generally lower than in conventional cigarettes. Regardless, multiple studies report that experienced E-cig users often attain serum cotinine concentrations that are similar to those levels found in cigarette smokers, and thus our exposure mirrored these E-cig users. Our E-cig exposure utilized a chamber. Despite a relatively high air-exchange rate, this exposure system does not preclude other routes of exposure in addition to inhalation, such as ingestion or absorption. Finally, this model only investigated the pulmonary effects after 2 wk exposures to a single E-cig brand/flavor/nicotine concentration. Our choice of menthol E-cigs may not be representative of other flavors. Menthol flavored E-cig liquids are moderately cytotoxic compared to other flavors [43], and there is little evidence to suggest that mentholated cigarettes are more or less toxic than other cigarettes. However, menthol suppresses nicotine metabolism [45], and thus may have health impacts that are not observed with other flavors. We partially addressed this limitation by exposing a group of mice to traditional E-cig vapor, and we observed similar effects on inflammatory cell number and *ex vivo* bacterial clearance as compared to menthol E-cig vapor. Future studies will further dissect the roles of different E-cig additives, flavorings, nicotine levels, propylene glycol/glycerin mixtures, vapor delivery devices, etc on pulmonary response in a longer exposure regimen. Future studies will also further explore the mechanism by which E-cig exposure impacts bacterial and viral infection.

In conclusion, E-cig exposure results in immunomodulatory effects that are similar to those observed after exposure to cigarette smoke. Since bacterial and viral exacerbations are major drivers of COPD disease progression, this study raises a concern that COPD patients who switch from cigarettes to E-cigs may not observe substantial improvement in their disease progression. Furthermore, popularity of E-cigs among teenagers is rapidly rising, which may lead to an emerging threat to public health with regards to recurrent bacterial or viral infections. Despite the common perception that E-cigs are safe, this study clearly demonstrates that E-cig use, even for relatively brief periods, may have significant consequences to respiratory health in an animal model; and hence, E-cigs need to be tested more rigorously, especially in susceptible populations.
